# Competitive programming participation rates: an examination of trends in U.S. ICPC regional contests

**DOI:** 10.1007/s44217-023-00034-1

**Published:** 2023-03-21

**Authors:** Jeremy J. Blum

**Affiliations:** Pennsylvania State University, Harrisburg, Harrisburg, PA USA

**Keywords:** Contest programming, Trends in contest participation, Student recruitment and retention, Contest problem set design

## Abstract

A wide range of benefits have been posited from participation in competitive programming contests. However, an analysis of participation in north American regional contests in the International Collegiate Programming Contest (ICPC) shows that participation in these contests is sharply declining, coinciding with the COVID-19 pandemic. Moreover, prior to the pandemic, while the number of teams participating in regional contests was increasing, the number of institutions sending teams to these contests was declining. We find several statistically significant correlations that may underscore structural reasons for this trend. Consistent participation in contests and the number of teams per institution sent to a contest both are correlated with likely participation in future contests. On the other end of the spectrum, institutions sending a team to a contest for the first time in 3 years were much less likely to return in the next year. For this category of teams, if a team is unable to solve any problems in the contest, the institution is significantly less likely to send a team in the next year. Many of these contests have very challenging problem sets, and consequently, have many teams that fail to solve any problems. This result suggests that structuring the problem sets to increase the likelihood that most teams successfully complete problems would broaden participation in these contests.

## Introduction

Despite the pedagogical benefits of programming contests, U.S. student participation remains low, particularly for students from under-represented groups [1, 2]. Researchers have suggested a range of potential barriers to participation, including institutional challenges in creating a programming team system and student perceptions of the utility of contests [1, 3].

This work analyzes trends in participation in north American regional ICPC contests. Participation in these contests declined significantly due to the COVID-19 pandemic. Even before the pandemic, there was a decline in the number of institutions sending teams to these contests, even as the number of teams overall increased. Understanding the root causes of these trends is critical to bring the benefits of contest participation to as broad a population as possible.

One goal for contest problem set developers is to create a challenging problem set that clearly differentiates between the top teams, since only a portion of these top teams advance to the next round of the competition. On the other hand, a consequence of a very challenging problem set is that a large number of teams may have limited success in the course of the 5-h contest. Indeed, we find that in more than a quarter of the ICPC North American contests, more than twenty percent of the teams fail to solve any challenges.

The analysis presented here suggests that the goal of a challenging problem set be balanced with one that allows some success for all teams. An exploratory data analysis and a logistic regression model of contest standing data found that the previous contest participation, number of teams in current competition, and success in the problem set are all positively correlated with the likelihood of an institution participating in the next contest. For example, for an institution sending a single team to the first contest in at least 3 years, success in solving at least one problem increased the likelihood of a return by almost 50%. This analysis suggests that problem set design could be a significant catalyst in broadening contest competition. As an institution’s teams experience success in an initial contest, they are likely to return for future contests, and repeated contest participation increases the likelihood of further participation.

## Related work

Programming contests hold promise of improving student learning outcomes and employment opportunities. Previous research, for example, has identified benefits, including improving student understanding of core concepts, building teamwork skills, and preparing students for technical interviews [1, 4]. Despite these benefits, participation is low, particularly among teams from the United States. For example, in the ICPC, “participation in North America lags behind that of the other ICPC super-regions, particularly Europe and Asia” [1]. In the 2020 IEEEXtreme global programming contest, 72 teams entered from the United States, compared to 176 teams from China, and 728 teams from India [5].

The need to better understand the potential for these contests is long-standing. Verhoeff lamented that “many academics at best tolerate CS competitions at universities. How many publications that address the special issues surrounding student competition have you seen in well-known academic journals?” [6] While there has been research since then on barriers to contest participation, our understanding of these barriers remains incomplete.

Some of this research has focused on institutional barriers to programming contest participation. Bloomfield and Sotomayor, for example, presented guidance for contest coaches to develop resources and training for team members [1]. On the other hand, other research has focused on student perceptions as a barrier to participation. Raman et al., for example, surveyed student contest participants in India and found that the most important factors for participation included peer influence and perceptions that participation would improve problem-solving abilities and lead to better employment opportunities [3].

Researchers have speculated that the perception of utility, enjoyment, and relevance is driven by the design of contest problem set. Boersen and Phillips posit that having a range of problem difficulty is critical [7]. They reason that beginning students experience success which can lead them to participate in subsequent contests, and the contests can provide motivation to improve their analytical skills over time. Our analysis supports this assertion.

The context of the contest questions has also been suggested as a potential barrier, particularly for some groups. Armoni posits that one factor is the focus of contests on programming as opposed to problem-solving, which may be a limiting factor, particularly for female participants [4].

Participation in contests among traditionally underrepresented groups lags significantly behind the general population. Fisher and Cox suggest the importance of problem context for appealing to a wide audience [2]. They suggest, for example, that stories focusing on sports may not appeal equally to all genders. A real-world context with broad appeal does appear to be important. One of the barriers that Raman et al. identified in their survey was the perception that the problems involved imaginary algorithmic puzzles with little applicability to the real world [3].

It is worth noting that some researchers have raised concerns about the pedagogical benefits of programming contests. For example, the evaluation of teams using black-box approaches based on submission time, encourage coding artifacts where code readability and documentation suffer [8]. Others have also agreed and noted that creativity also suffers when the solution approaches are dictated by the problems [9, 10].

## Methods

The data used for analysis was gathered from a variety of sources beginning with the ICPC results site for the North American Regional Contest.^1^ Missing contest results were added where possible from a variety of sources, as detailed later. The data was cleaned to remove duplicate records and standardize the institution names. The source data was aggregated by institution and year to create a dataset for analysis. The dataset was then used to examine trends in participation and correlations between likelihood that an institution returns to the next year’s contest and attributes derived from the scoreboard data.

The data provided by the ICPC results site includes multiple types of contests beyond the standard yearly regional contests, including online qualifying contests and special one-off contests. Only the North American regional contests were considered for this analysis. For these contests, Table 1 lists the attributes for each team are reported in the final standings data.

**Table 1:** Attributes in ICPC results data

Field	Description
Rank	Final ranking for the team in the contest
Team name	Name for the team
Institution name	Name of the university or college for the team
Problems solved	Number of problems correctly solved during the contest
Total time	The cumulative time used to solve problems. This includes a sum of the submission times for all successfully submitted problems. In addition, for any problem that is correctly solved, the total time includes a 20-min penalty per incorrect submission to this problem
Last time	The last time that a correct solution was submitted

There were a number of data quality issues in the results pages, including duplicate entries, missing values, and variations in institution names. Prior to data aggregation, these issues were addressed.

The ICPC results pages include results that contain duplicate entries, where, for example, the results for each team are reported two, three, or even four times. These duplicate entries were removed. Some contest results were missing, and in this case, the results were found on a mirror site hosted at Baylor University.^2^

In addition, many results were missing data including some combination of the Problems Solved, Total Time, and Last Time fields. An Internet search for final standings located this missing data for many of the contests.^3^^,^^4^^,^^5^^,^^6^^,^^7^ The final source data included records from regional contests between 2000 and 2021, inclusive, with results from 22,851 teams.

An examination of the institutions revealed variations in the names recorded in the results. A set of approximately 85 rules were created to standardize the institution names.

After addressing these data quality issues, the contest results were aggregated by institution, year, and contest. Twenty-two out of the more than 8000 observations were for institutions that participated in multiple regional contests in the same year. Since some of the attributes related to aspects of a particular contest, these records were held out of the final dataset. Table 2 lists the attributes that were created in the final dataset.

**Table 2:** Attributes after aggregation by institution, region, and year

Field	Description
Institution name	Name of the university or college
Region	ICPC region name
Year	Contest year
Participate	Did the institution send teams to a regional contest in the next year
Past 3 years	How many times did the institution send teams to contests in the past 3 years (including the current contest)
Team count	How many teams did the institution send to the current contest
Max solved	The maximum number of problems solved by a team from this institution in the current contest
Average solved	The average number of problems solved by a team from this institution in the current contest
Min solved	The minimum number of problems solved by a team from this institution in the current contest
Max total time	The maximum total time field for a team from this institution in the current contest
Average total time	The average total time field for a team from this institution in the current contest
Min total time	The minimum total time field for a team from this institution in the current contest
Max last time	The maximum last time field for a team from this institution in the current contest
Average last time	The average last time field for a team from this institution in the current contest
Min last time	The minimum last time field for a team from this institution in the current contest
Contest max solved	The number of problems solved by the winning team at this contest
Contest average solved	The average number of problems solved by a team at this contest
Contest percent failed	The percentage of teams that failed to solve any problems at this contest

## Factors correlated with future contest participation

The analysis of factors correlated with future contest participation began with an exploratory data analysis of the aggregated scoreboard data. In addition to identifying trends in contest participation, this analysis identified a set of attributes that are correlated with future participation. The most correlated attributes are then used to create a logistic regression model that predicts the likelihood of participation. The model results provide insights into strategies for broadening contest participation.

### Exploratory data analysis

**Fig. 1:**
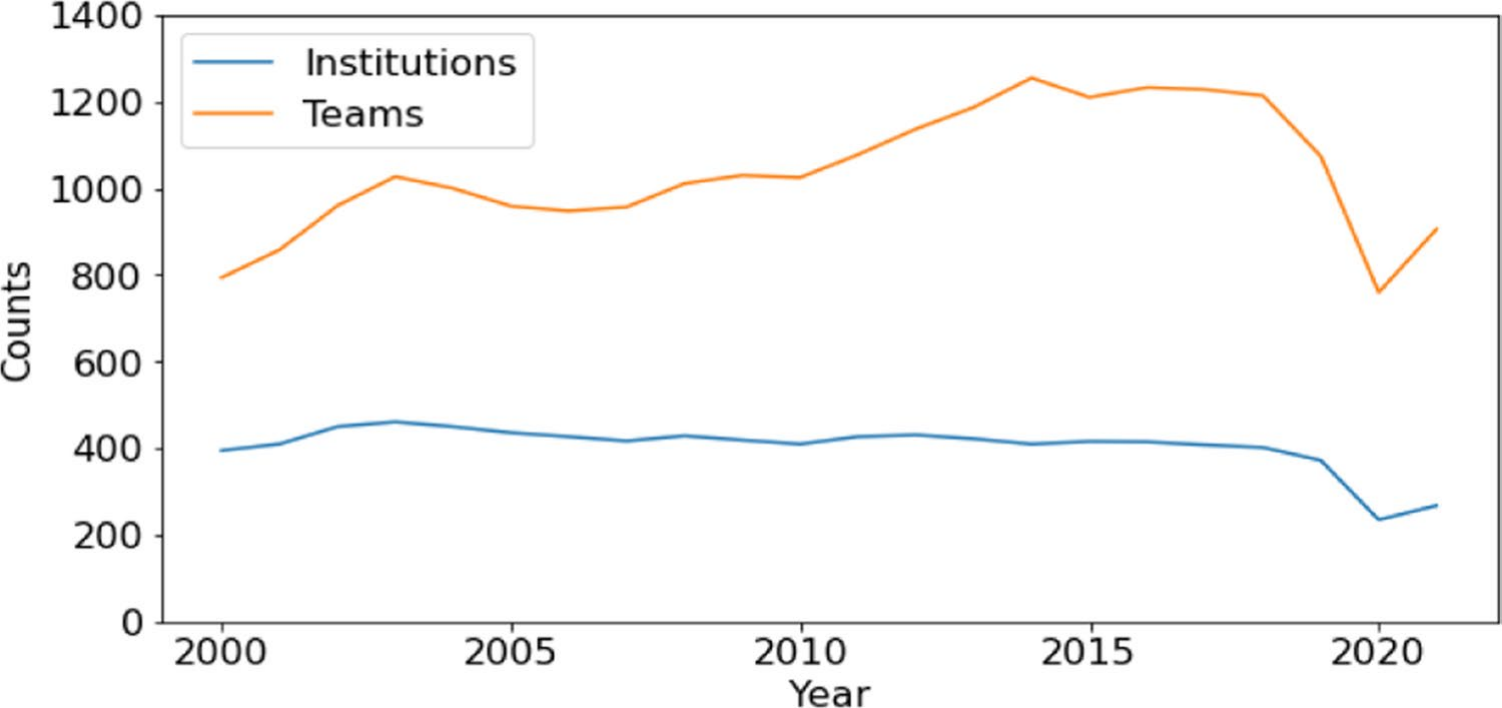
Number of teams and institutions participating in ICPC North America Regional Contests, 2000–2021

The COVID-19 pandemic had a clear negative impact on contest participation. Figure 1 shows trends in the number of teams participating in North America ICPC regional contests, as well as the number of institutions sending teams to these contests. Both these counts experienced a significant drop in 2020 due to the pandemic. In 2021, both the number of teams and number of institutions experienced a rebound, although they both are still short of pre-pandemic levels.

Prior to the pandemic, the number of teams participating in these contests experience an increase from 794 in 2000 to 1074 in 2019, with a high of 1255 in 2014. While the number of teams was increasing, the number of institutions fell. The number of institutions experienced a high of 461 in 2003 and had fallen to 372 in 2019.

To eliminate this effect of the pandemic on participation, we limited data to contests between 2002 and 2018. The attribute Past 3 years meant that the first 2 years of contests could not be included. Similarly, restricting the data to pre-pandemic period, meant that the largest value for the Year field would be 2018, so that the Participate attribute for this Year would correspond to participation in the 2019 pre-pandemic contests.

In order to explore what factors may be correlated with participation in the next contest, we began by examining the correlation coefficient of all of the attributes listed in Table 2. The variables with the largest correlation to Participate are listed in Table 3. Note that the Max Total Time and Max Solved attributes are themselves highly correlated. Of the two variables, we chose to focus on Max Solved because it is easier to interpret. Large values in Max Solved indicate more success in a contest. Large values of Max Total Time, on the other hand, may occur in two distinct scenarios. This attribute will be large if multiple problems are solved. However, in less common cases, it may also be large if a team solves only one problem but takes the entire contest to do so.

**Table 3:** Attributes with largest correlation with participation in next contest, 2000–2018

	Participate	Past 3 years	Team count	Max total time	Max solved
Participate	1	0.235	0.195	0.176	0.175
Past 3 years		1	0.252	0.160	0.155
Team count			1	0.359	0.335
Max total time				1	0.853
Max solved					1

**Fig. 2:**
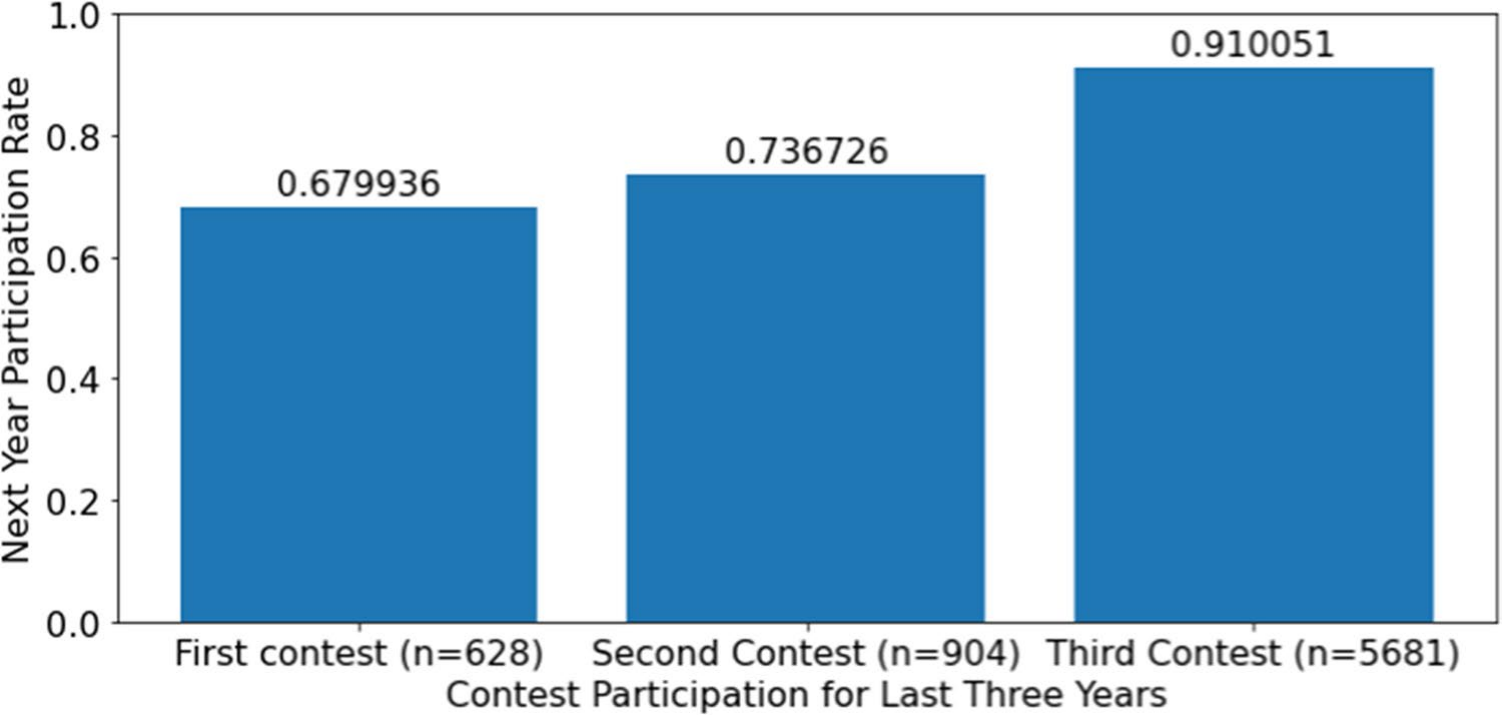
Return rate as a function of the number of contests in last 3 years, 2002–2018

Figure 2 plots the rate at which institutions returned to the next year’s contest based on how many regional contests they participated in during the last 3 years. Teams that participated in all three competitions returned 91.0% of the time, where teams that participated in their first contest returned just 68.0% of the time. The average number of contests for institutions that returned was 2.76 and average number of contests for institutions that did not return was 2.33. This difference is statistically significant (*p*-value of 0.0000).

**Fig. 3:**
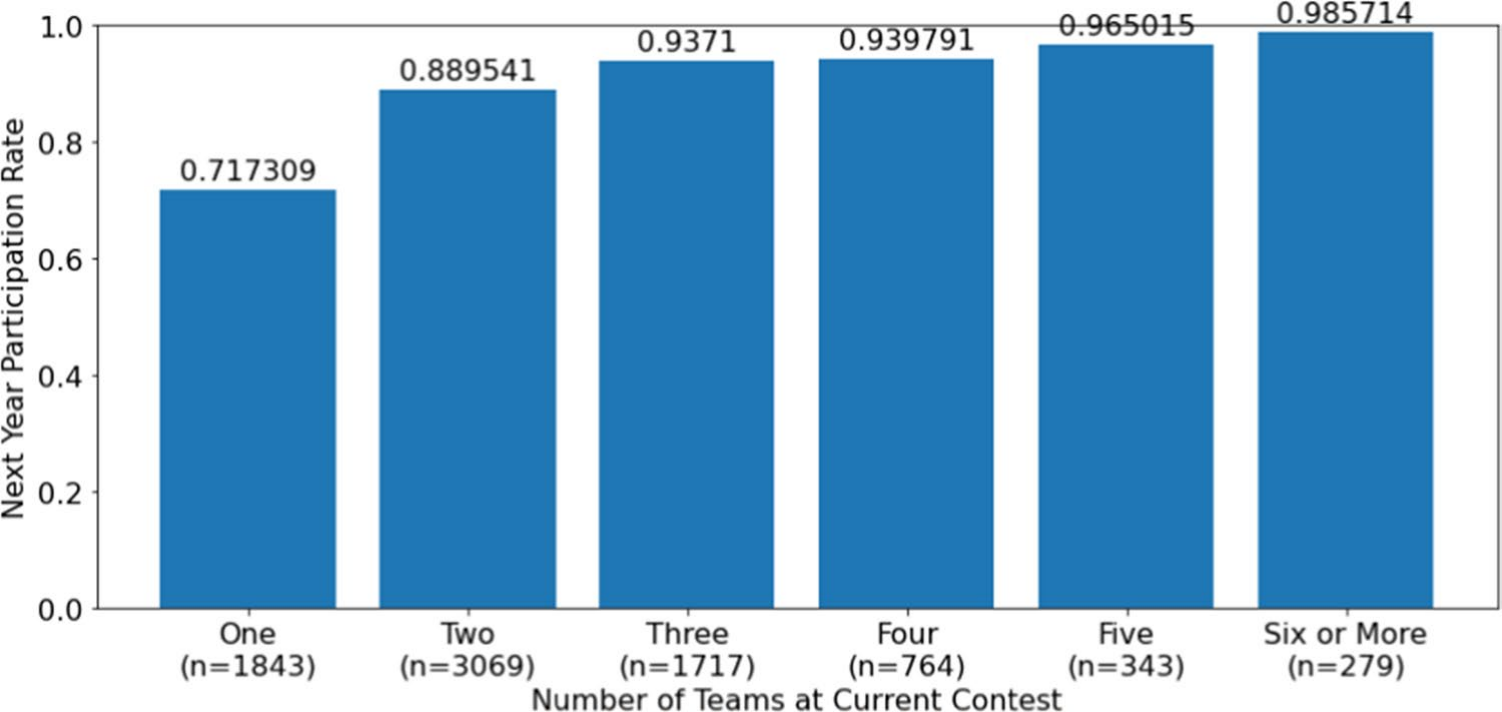
Return rate as a function of the number of teams sent to the current contest, 2002–2018

Figure 3 plots the rate at which institutions returned to the next year’s contest based on how many teams they sent to the current contest. There is a large jump in the return rate for institutions that sent two teams rather than just one (89.0% vs. 71.7%). The average number of teams for institutions that returned was 2.67 and average number of teams for institutions that did not return was 1.77. This difference is statistically significant (*p*-value of 0.0000).

**Fig. 4:**
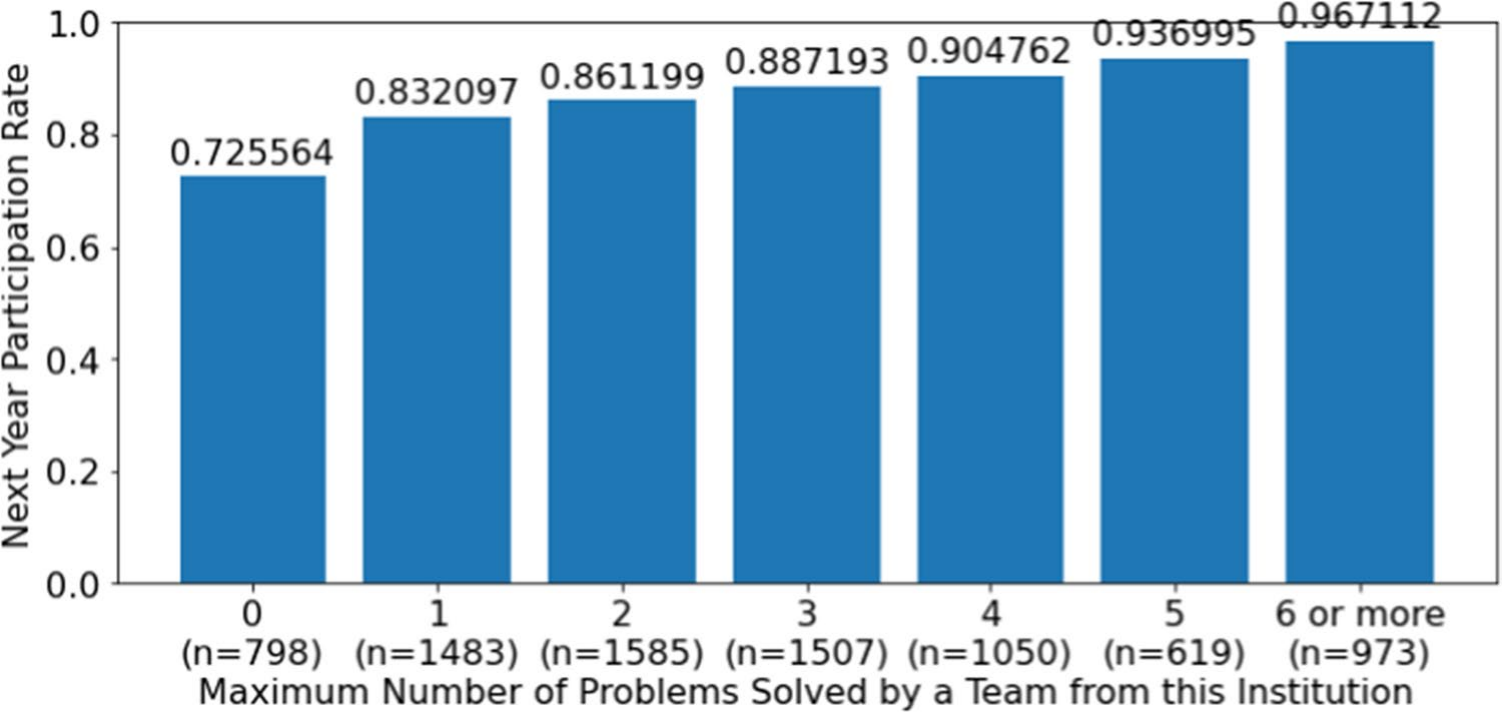
Return rate as a function of the maximum number of problems solved by any team from a given institution, 2002–2018

Figure 4 plots the rate at which institutions returned to the next year’s contest based on how many problems were solved by their most successful team in the current contest. Institutions whose best team was unable to solve any problems returned 72.6% of the time. This return rate jumped to 83.2% for institutions whose best team solved one problem, and it continued climbing as the number of problems solved increased. The average of the Max Problem Solved variable for institutions that returned was 2.41 and average number of contests for institutions that did not return was 1.54. This difference is statistically significant (*p*-value of 0.0007).

### Logistic regression model

Next a logistic regression model with Participate as the dependent variable with Last 3 years, Team Count, and Max Solved as independent variables. As shown in Table 4, the model provided a significantly better fit than an intercept-only model, indicating that we can reject the null hypothesis that there is no relationship between the independent and dependent variables. Moreover, as shown in Table 5, each of the independent variables was found to be statistically significant with *p*-values of 0.000.

**Table 4:** Logistic regression model fit results

Log-likelihood	− 2470.3
Log-likelihood null model	− 2808.3
Log-likelihood ratio *p*-value	3.442e − 146

**Table 5:** Logistic regression model coefficients

	Coef	SE	Z	*P* >|z|	[0.025	0.975]
Intercept	− 1.1644	0.132	− 8.795	0.000	− 1.424	− 0.905
Past 3 years	0.5819	0.049	11.973	0.000	0.487	0.677
Team count	0.4912	0.043	11.507	0.000	0.408	0.575
Max solved	0.2027	0.022	9.139	0.000	0.159	0.246

The interpretation of the regression coefficients in Table 5 conforms to the exploratory data analysis. The coefficients in the logistic regression model represent the expected change in the log-odds of participation based on each of the explanatory variables. Thus, for a coefficient $\beta$, ${e}^{\beta }$, provides the change in odds of participation for a one-unit change in the explanatory variable. For example, for each additional year that university that has participated in the last 3 years, there is a 78% increase in the odds of participating next year (1.78 = e^0.5819^). Similarly, each additional team sent to a contest increases the odds of participating next year by 63% (1.63 = e^0.4912^). Finally, each problem solved by the best team sent by an institution increases the odds of participating next year by 22% (1.22 = e^0.2027^).

### Implications for broadening participation

The results from the exploratory analysis and regression model support the importance of previous research focusing on strategies and resources for developing robust programming teams at an institution. Having a strong program would tend to create consistency in attendance at contests, the ability to send multiple teams to contests, and better success for teams at the contests.

**Fig. 5:**
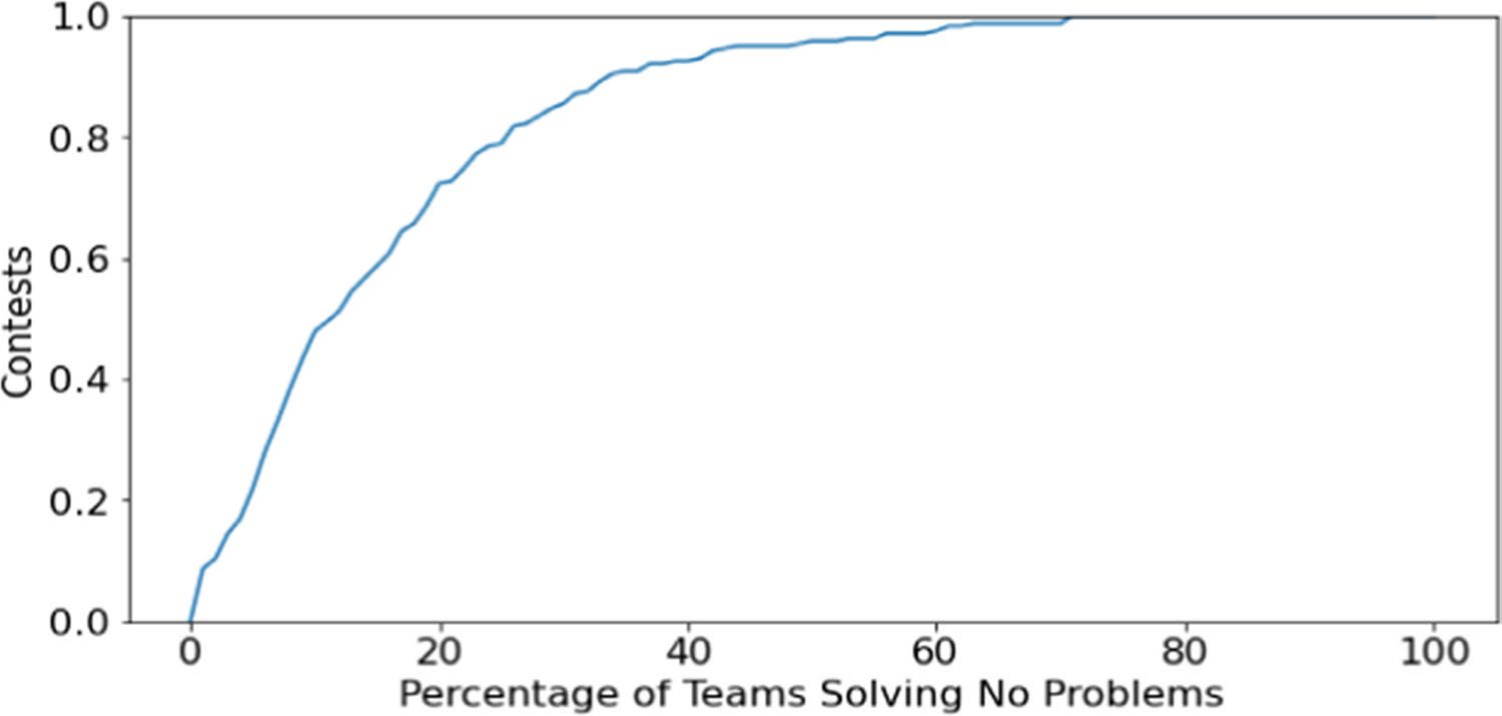
Empirical cumulative distribution function of contests and percentage of teams in contest solving no problems, 2000–2019

There are also implications for contest problem set design. There are a number of contests in which there is a significant percentage of teams that are unable to solve any problems. Figure 5 shows an empirical cumulative distribution function for the percentage of teams solving none of the problems in the ICPC regional contests. Ten percent or more of teams solve no problems in 52.1% of contests, 20% or more of teams solve no problems in 27.7% of contests, and 50% or more of the teams solve no problems in 4.13% of contests. Their lack of results occurs despite their effort in their five-hour contest.

**Fig. 6:**
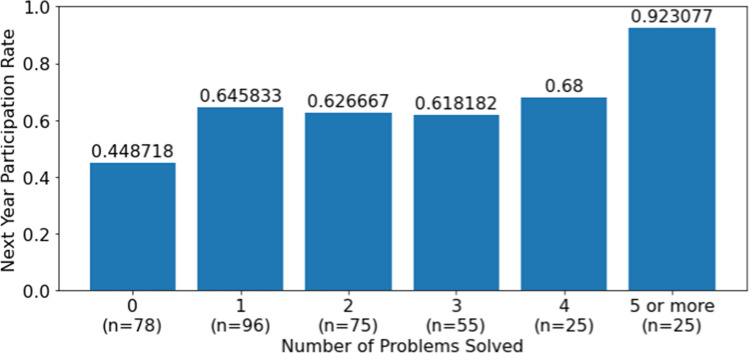
Return rate for institutions participating in first contest in at least 3 years, and sending only one team, as a function of the number of problems correctly solved, 2002–2018

This lack of success seems to be particularly problematic for institutions that may be trying to launch a programing team system. Figure 6 shows the return rate for institutions that are sending one team to a contest, when they have sent no teams in the previous two contests. As shown in the figure, these institutions return only 44.9% of the time if their team is not able to solve any problems. If the team can solve one problem, the return rate jumps to 64.6%. Overall, if their team solves 1 or more problems, the return rate is 66.2% for these institutions.

These results suggest that the contest problem set design could help broaden participation in programming contests. If the problem sets are designed to increase the likelihood that almost all teams solve some problems, institutions, which are seeking to establish a programming team system, would be more likely to return to future contests, perhaps even with more teams. The analysis suggests that this return would create a positive feedback loop, with repeated attendance and more teams further increasing the likelihood of future participation.

## Conclusions and future work

Previous research has suggested a range of benefits arising from participation in programming contests. Nonetheless, participation in contests lags, particularly in North America. Analysis found that the COVID-19 pandemic caused large reductions in participation. Even prior to the pandemic, while the number of teams grew between 2000 and 2018, the number of institutions sending teams decreased.

A logistic regression model was constructed to predict the likelihood that an institution will return to a contest based on scoreboard-related attributes. The analysis suggests that the following are all positively correlated with continued participation: consistency in contest attendance, the number of teams sent to the current contest, and the number of problems solved by an institution’s best team. For institutions sending their first team to a contest in at least 3 years, being able to solve at least one problem increased the likelihood that the institution will return by almost 50%.

These results have important implications for contest problem set design. The model suggests that organizers could broaden participation in programming contests by designing the problem set to increase the likelihood that ensure that all teams experience success in the contest. Success in an early contest appears likely to create a positive feedback loop. The success makes continued participation more likely, and continued participation further increases the likelihood of a return to future contests.

In future work, we would like to look at an expanded dataset that can support models that include a larger range of explanatory factors, beyond team performance, and their effect on aggregate participation rates. An example of these factors would be the prerequisite knowledge needed to solve the problems in a problem set. In addition, we would like to consider factors that measure the extent to which scaffolding is provided to help contestants structure and debug their solutions, for example, whether participants are given information about partial correctness of their problems.

## Data Availability

The location of the data is provided in footnotes throughout the manuscript.

## Footnotes

1 ICPC, Regional Results, https://icpc.global/regionals/results. Last Accessed: 8/1/2022.

2 ICPC, Regional Results (Baylor Mirror), https://icpc.baylor.edu/regionals/results/. Last Accessed 6/1/2022.

3 ICPC, Regional Results (Baylor Mirror), https://icpc.baylor.edu/regionals/results/. Last Accessed 6/1/2022.

4 Kattis, (2016) Mid-Central USA Programming Contest 2016 Standings, https://mcpc16.kattis.com/contests/mcpc16/standings, Last Accessed: 8/3/2022.

5 W.D. MacEvoy, (2005–2010) Rocky Mountain Regional Scoreboard, https://org.coloradomesa.edu/~wmacevoy/rmrc/2010/scoreboard_byrank.html, Last Accessed: 8/3/2022.

6 ICPC Southeast USA Regional Programming Contest, (2007–2009), Standings, http://seusa.vanb.org/ser2009/standings.html, Last Accessed: 8/3/2022.

7 ICPC East Central NA Regional Programming Contest, (2003–2008), Standings, https://icpc-ecna.ysu.edu/PastResults/2008/standings.html, Last Accessed: 8/3/2022.

## Code availability

Available upon request.
